# Supplementary material for: Quality of Oreochromis niloticus and Cynoscion virescens fillets and their by-products in flours make for inclusion in instant food products
Source: PLoS One. 2023 Feb 17;18(2):e0279351. doi: 10.1371/journal.pone.0279351 (PMC9937477; doi:10.1371/journal.pone.0279351)
Supplement: Attachment — Submitted filename: Response to Reviewers.docx [file pone.0279351.s001.docx]

**-Question and** Answers/Justifications

**All other requests for adjustments were made in the text.**

- lines 128 to 139: were these ingredients used in these proportions as in the previous work?

Not. The ingredients and quantities were proposed by the researchers.

**-Table 3: Why was it chosen to perform the analysis of the amino acid profile only for backbone flours?**

Due to the high cost, it would not possible to make amino acids from all flours, so we chose to present the one with the best result in terms of protein content.

**-Line 394: The authors report that a temperature of 90°C was used for the dehydration of the flours. However, it is known that high temperatures during drying lead to several irreversible biological or chemical reactions, in addition to structural, physical and mechanical changes. Several works that developed fishmeal used milder temperatures. What led the authors to use 90°C?**

As the carcasses and heads were cooked for 60 minutes, for the preparation of the flours, it was decided to use a higher temperature in order to reduce the dehydration time and to reduce the chance of any contamination during the milling process of the raw materials. With dehydration at a higher temperature, it can reduce or eliminate the possibility of contamination by microorganisms., in addition to comparing the results obtained with the literature? Comparing the results obtained, it can observed that many parameters were superior to those in the literature.

Competing interests: The authors have declared that no competing interests exist.

Data Availability: All relevant data is in the paper and its Supporting information files.

Funding: This study received financial support from CAPES/Brasil through Programa Nacional de Cooperação Acadêmica na Amazônia - PROCAD-AM (UNIR/UFAC/USP), for the granting of a postdoctoral scholarship to Jerônimo Vieira Dantas Filho.

Competing interests: The authors have declared that no competing interests exist.
